# Exploring the selective constraint on the sizes of insertions and deletions in 5' untranslated regions in mammals

**DOI:** 10.1186/1471-2148-11-192

**Published:** 2011-07-05

**Authors:** Chun-Hsi Chen, Ben-Yang Liao, Feng-Chi Chen

**Affiliations:** 1 Division of Biostatistics and Bioinformatics, Institute of Population Health Sciences, National Health Research Institutes, Zhunan, Miaoli County, 350 Taiwan; 2 Department of Life Science, National Chiao-Tung University, Hsinchu, 300 Taiwan; 3 Department of Dentistry, Chinese Medical University, Taichung, 404 Taiwan

## Abstract

**Background:**

Small insertions and deletions ("indels" with size ≦ 100 bp) whose lengths are not multiples of three (non-3n) are strongly constrained and depleted in protein-coding sequences. Such a constraint has never been reported in noncoding genomic regions. In 5'untranslated regions (5'UTRs) in mammalian genomes, upstream start codons (uAUGs) and upstream open reading frames (uORFs) can regulate protein translation. The presence of non-3n indels in uORFs can potentially disrupt the functions of these regulatory elements. We thus hypothesize that natural selection disfavors non-3n indels in 5'UTRs when these regulatory elements are present.

**Results:**

We design the Indel Selection Index to measure the selective constraint on non-3n indels in 5'UTRs. The index controls for the genomic compositions of the analyzed 5'UTRs and measures the probability of non-3n indel depletion downstream of uAUGs. By comparing the experimentally supported transcripts of human-mouse orthologous genes, we demonstrate that non-3n indels downstream of two types of uAUGs (alternative translation initiation sites and the uAUGs of coding sequence-overlapping uORFs) are underrepresented. The results hold well regardless of differences in alignment tool, gene structures between human and mouse, or the criteria in selecting alternatively spliced isoforms used for the analysis.

**Conclusions:**

To our knowledge, this is the first study to demonstrate selective constraints on non-3n indels in 5'UTRs. Such constraints may be associated with the regulatory functions of uAUGs/uORFs in translational regulation or the generation of protein isoforms. Our study thus brings a new perspective to the evolution of 5'UTRs in mammals.

## Background

Insertion and deletion mutations (indels) frequently occur during the evolution of mammalian genomes [1-4]. Most of these indels are selectively neutral when they occur in noncoding genomic regions [5,6]. Meanwhile, indels indivisible by three (designated as "non-3n" indels, in contrast to "3n indels", indels divisible by three) were found to be underrepresented in coding sequences (CDS) [4,7]. This is because non-3n indels lead to frameshift in CDS (which frequently results in pseudogenization), while 3n indels do not. Therefore, non-3n indels in CDS have larger chances of being removed by natural selection. If reading frame preservation is the only evolutionary constraint on indel size, 3n indels and non-3n indels should have the same probability of being retained in noncoding regions. Indeed, a former study only found an overrepresentation of 3n indels in CDS, but not in noncoding sequences in the human genome [4].

**Figure 1:**
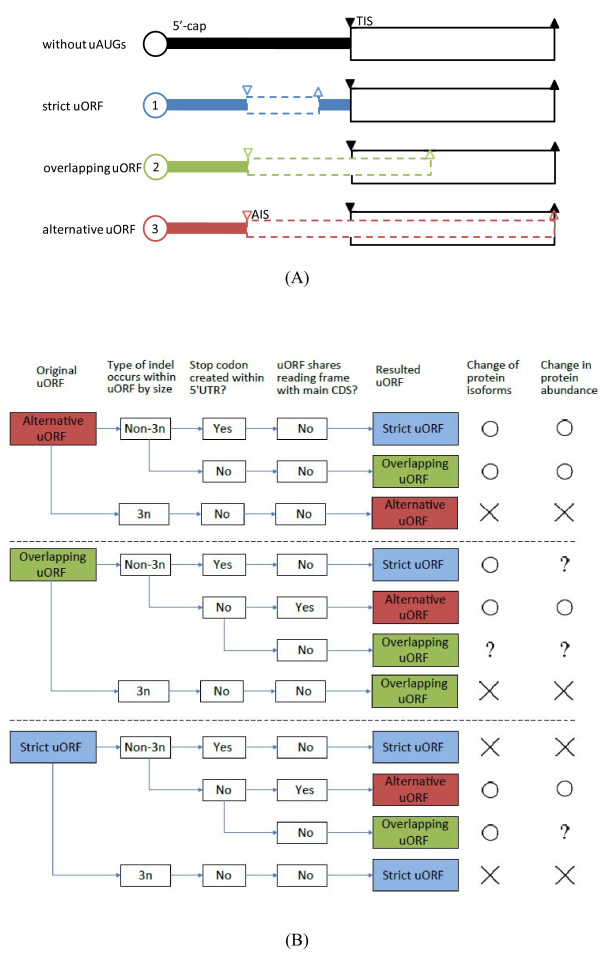
**Classification of upstream open reading frames (uORFs) and potential effects of non-3n indels when they occur between uAUGs and TISs**. (A) The open circle represents the 5'-cap structure of the transcript. The solid- and dashed-line open boxes represent the main coding sequences and the uORFs, respectively. The open and solid inverted triangles, respectively, indicate the locations of the uAUGs and the translation initiation sites (TIS). The open and solid triangles indicate locations of the stop codons of the uORFs and the main coding sequences. (B) Symbols "○", "×", and "?" represent that the protein isoforms or protein expression "is affected", "is not affected", and "uncertain", respectively.

Among noncoding sequences, 5'-untranslated regions (5'UTRs) are of particular interest because of their transient presence between transcriptome and proteome, and their regulatory effects on both transcription and translation [8-10]. The primary translational regulatory elements in 5'UTRs include translation initiation motifs [11], upstream start codons (uAUGs), and upstream open reading frames (uORFs) [12,13]. uORFs can be classified into three major categories [14]: (i) strictly upstream ORFs ("strict uORFs") -uORFs entirely located within 5'UTR; (ii) CDS-overlapping uORFs ("overlapping uORFs") - uORFs with their start codons located in 5'UTR and their stop codons located within the CDS; and (iii) alternatively translated uORFs ("alternative uORFs") -uORFs that start with an alternative initiation site of translation ("AIS", a potential translation initiation site located in 5'UTR), and share the same reading frame and the same stop codon with the main CDS (Figure 1A). Note that the protein products that include alternative uORFs are currently unidentified. Therefore, for extensively studied species such as human and mouse, AISs are less likely to be misannotated canonical translation start sites. The scanning model for translation [15] posits that a translation process begins with the binding of 40S ribosomal complexes onto the 5'-cap structure of an mRNA. The ribosomal complexes then slide from 5'-cap to 3' end base by base until they encounter the first AUG triplet of the mRNA and turn on the translation process. According to this model, different types of uORF have distinct effects on the translation of the main CDS: (1) strict uORFs compete for ribosomes, leading to reduced protein production of the main CDS [16]; (2) overlapping uORFs cause translation re-initiation downstream of the main start codon, resulting in N-truncation of the peptide coded by the main CDS [10,17]; they may also cause strong translational inhibition by facilitating ribosome skipping of the main start codon [10,18,19]; and (3) alternative uORFs result in the generation of N-extended peptides [17,20,21]. Notably, non-3n indels downstream of uAUGs can lead to interchanges between uORF types (Figure 1B), while 3n indels usually can not. Such interchanges may result in either changes of protein abundance (quantitative changes) or gain/loss of protein isoforms (qualitative changes) (Figure 1B). Considering that the level of protein translation is tightly constrained during evolution [22], most changes between uORF types are supposedly deleterious. Therefore, we hypothesize that in 5'UTRs, non-3n indels that occur downstream of uAUGs are subject to purifying selection.

To test our hypothesis, we develop the Indel Selection Index (ISI, see Methods) to examine whether the occurrence of non-3n indels between the first uAUGs and the translation initiation site ("TIS", also known as the main start codon) of the main CDS are selectively disfavored during the evolution of mammalian genes after primate-rodent divergence. Since non-3n indels may affect protein expression more seriously when they occur downstream of the uAUGs of alternative uORF and overlapping uORFs (Figure 1B), purifying selection on non-3n indels is expected to be particularly stringent in these regions, while relatively relaxed in the case of strict uORFs. Our results clearly support this hypothesis. This study thus offers a new perspective to the evolution of 5'UTRs, in that the sizes of indels can be subject to selective constraint in these genomic regions conditional on the presence of certain regulatory elements.

## Results

We compared human and mouse orthologous genes to examine whether non-3n indels are selectively constrained downstream of uAUGs. While the range of 5'UTRs varies in alternatively spliced mRNA isoforms, we used three different criteria to select one transcript for each gene for this analysis (see Methods): (1) a randomly selected transcript, (2) the transcript with the longest 5'UTR, and (3) the transcript with a "pure" 5'UTR (i.e. a 5'UTR that does not overlap with the CDS of any other splicing isoforms). These three different selection criteria have distinct biological implications. The first dataset assumes that for each gene, all of the isoforms are equally important to the organism. The assumption may not be true, but random sampling may fairly rule out potential sampling biases. The second dataset contains the largest number of uAUGs. However, some uAUGs in these 5'UTRs may overlap with the CDS of other isoforms of the same gene. In contrast, although the third dataset enables us to explore the selection pressure that works exclusively on 5'UTRs, only a small proportion of transcripts with biased properties can be included in the analysis. These transcripts tend to be the products of the genes with short 5'UTR, genes that are lowly expressed, or genes not alternatively spliced, although > 90% of human genes have multiple isoforms [23]. Given the distinct properties of selected transcripts in the three datasets, we reason that if our hypothesis is correct, all three datasets will yield consistent results.

According to our criteria, more than 45% human genes and 41% mouse genes have at least one uORF (Table 1). These percentages are similar to those reported in previous studies [16,24]. Next, we divided the transcripts of each dataset into four subgroups for comparisons: (1) transcripts without uAUGs (G_0_); (2) transcripts with only alternative uORF(s) (G_a_); (3) transcripts with only strict uORF(s) (G_s_; their uAUGs are designated as "SuAUGs"); (4) transcripts with only overlapping uORF(s) (G_v_; their uAUGs are designated as "VuAUGs"). Transcripts with multiple types of uORFs were excluded from this study for simplicity.

**Table 1:** Transcripts of human-mouse orthologous genes analyzed in this study

		**No. of genes (%)**^**b**^					
		
Type	**Class **^**a**^	Randomly-selected 5'UTR		Longest 5'UTR		Pure 5'UTR	
				
		Human	Mouse	Human	Mouse	Human	Mouse
Without uORF	G_0_	3,265 (54.0%)	3,560 (58.9%)	2,701 (46.6%)	3,153 (54.4%)	3,144 (55.2%)	3,368 (59.1%)
	G_a_	73 (1.2%)	61 (1.0%)	99 (1.7%)	76 (1.3%)	38 (0.7%)	40 (0.7%)
Single uORF type	G_s_	1,558 (25.8%)	1,456 (24.1%)	1,564 (27.0%)	1,487 (25.6%)	1,638 (28.7%)	1,523 (26.7%)
	G_v_	401 (6.6%)	385 (6.4%)	356 (6.1%)	333 (5.7%)	380 (6.7%)	345 (6.1%)
Multiple types of uORF		749 (12.4%)	584 (9.7%)	1080 (18.6%)	751 (12.9%)	500 (8.8%)	424 (7.4%)
Total		6,046	6,046	5,800	5,800	5,700	5,700

^a ^G_0_, G_a_, G_s_, and G_v _indicate transcripts without uAUGs, with AISs, with SuAUGs, and with VuAUGs, respectively.

^b ^For each gene, only one transcript is selected (a randomly selected transcript, or the one that has the longest 5'UTR or pure 5'UTR).

To evaluate the evolutionary constraints on indel lengths in different parts of a 5'UTR, we developed the Indel Selection Index ("ISI"; see Methods). In brief, ISI measures the probability of observing a higher frequency of non-3n indels downstream of a uAUG (or a reference point), as compared to the 5'UTR region upstream of the uAUG (or reference point). In other words, a small ISI indicates a depletion of non-3n indels downstream of a certain 5'UTR position. The use of ISI thus controls for the properties potentially specific to 5'UTRs. We first analyzed the ISI distribution of the G_0 _transcripts, where the ratios of non-3n to 3n indels are expected to be approximately equal between the "upstream" and "downstream" regions to a given position of the 5'UTRs. We assigned a reference point to each of the transcript and shifted the point from 10% to 90% of the 5'UTR lengths (with intervals of 10%) from the cap to obtain the ISI values. ISI values of the G_0 _transcripts vary with the reference point position (Additional file 1). For both human and mouse, the median ISI values remain approximately equal when the reference point is located at 30~70% of the 5'UTR lengths from the 5' cap, but drop toward both ends of 5'UTR. Therefore, controlling the ratio of upstream/downstream length is necessary when analyzing the ISI values. We then compared the ISI values of uAUG-containing transcripts (G_a_, G_s_, and G_v_) with those of G_0 _transcripts with corresponding upstream/downstream ratios. Note that the uAUG-containing transcripts may have multiple uAUGs of the same type. In such cases, we used the first uAUG from the cap as the reference point.

**Figure 2:**
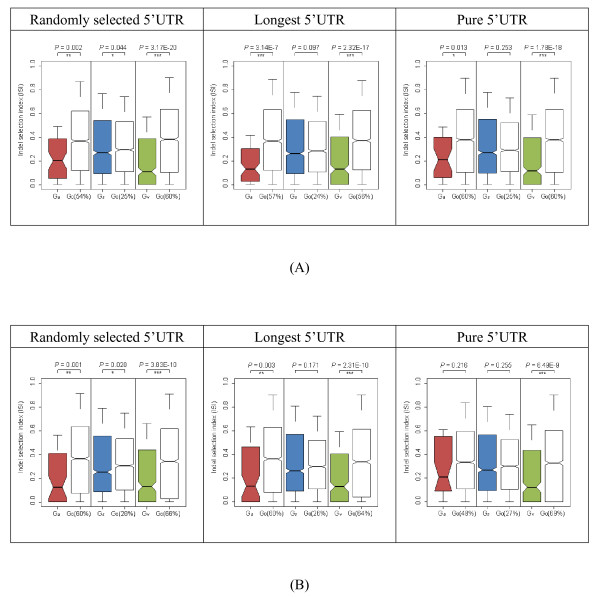
**Distributions of ISI values of (from left to right) (G_a _VS. G_0_), (G_s _VS. G_0_), and (G_v _VS. G_0_) for (A) human and (B) mouse**. The numbers in the parentheses following G_0 _indicate the median distances of the uAUGs from 5' cap in terms of percentage of 5'UTR length in the non-G_0 _transcripts. These proportions of length are referenced to determine which G_0 _distributions to use in the comparisons. The *P *values of pair-wise differences (calculated by using the Mann-Whitney *U *test) are shown at the top. The symbols "*", "**", and "***" represent 0.01 ≦ *P *< 0.05, 0.001 ≦ *P *< 0.01, and *P *< 0.001, respectively.

We found that G_v _transcripts consistently show significantly lower ISIs than the corresponding G_0 _transcripts in all of the six comparisons (*P *< 10E-8, all of the *P *values for the ISI value comparisons were estimated by using the Mann-Whitney *U *test, Figure 2). Meanwhile, the G_a _transcripts have significantly lower-than-expected ISIs (*P *< 0.05) in five of the six comparisons. The only exception lies in mouse transcripts with pure 5'UTRs (*P *= 0.216), although the median ISI of the G_a _transcripts is lower than that of G_0 _for this dataset. The lack of statistical significance may have resulted from the relatively small sample sizes (only 33 indel-containing G_a _transcripts are available for both human and mouse). In comparison, in only two of the six comparisons (randomly selected 5'UTRs in both human and mouse) do G_s _transcripts have significantly lower ISIs than expected (*P *< 0.05). We noticed that the sizes of indels might change with alignment tools [25], which could potentially affect our results. Therefore, we used Pecan [26], another alignment tool known for its accuracy, to re-align the human-mouse orthologous sequences and obtained similar results (Additional file 2).

Notably, the locations of reference points of the corresponding G_0 _transcripts actually differ significantly among G_a_, G_s_, and G_v_. In view of the variations of ISI values with different reference point locations (Additional file 1), the comparisons among the three transcript groups appear unfair. Particularly, all of the G_s _transcripts are compared against the G_0 _transcripts with their reference points located at ~25% from the cap, whereas the percentages for G_a _and G_v _fall between 54%~69% (Figure 2). To address this issue, we divided the G_v _transcripts into three equal-sized groups according to the relative positions of their uAUGs (we did not perform the analysis for G_a _because of its small sample size). As shown in Additional file 3 the first G_v _subgroups (G_v__1) were compared against G_0 _transcripts with reference points located at 14%~16% from the cap for human, and 24%~26% for mouse. These G_v _transcripts have uAUGs located closer to the cap than their G_s _counterparts, and they still have ISI values significantly lower than the corresponding G_0 _transcripts. Similar results are also observed for G_v__2 (Additional file 3). G_v__3 transcripts show a similar trend, although the differences in ISI values are statistically insignificant, possibly due to reduced sample sizes.

**Figure 3:**
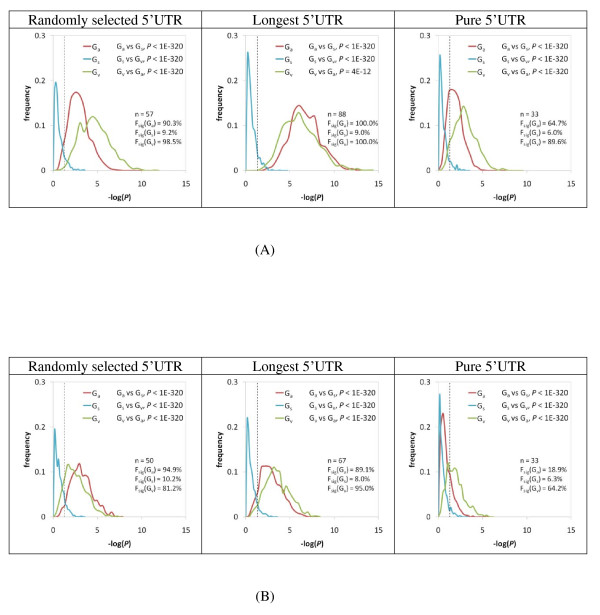
**Distributions of the *P *values in different ISI comparisons by controlling the sample sizes (indicated by "n") of G_s _and G_v _transcripts to be the same as those of the G_a _transcripts**. One thousand times of re-sampling with replacement was performed for each transcript subgroup to derive the *P *value distributions. The *P *values were estimated by using the Mann-Whitney *U *test. The dashed line indicates *P *= 0.05 (or -log (*P*) = 1.301). "F_sig_" indicates the fraction of *P *values in the distribution that is smaller than 0.05. The statistical significance between *P *value distributions of different transcript subgroups was estimated by using the Kolmogorov-Smirnov test and shown in the upper right corner of each panel.

Apparently, non-3n indels are significantly more depleted when occurring downstream of AISs and VuAUGs than SuAUGs. However, since G_a_, G_v_, and G_s _have different sample sizes, we are interested in comparing how the ISIs of G_a_, G_v_, and G_s _deviate from the expected values when controlling for the difference in sample size. We performed a bootstrap simulation (with 1,000 re-samplings with replacement) for each of the six comparisons by reducing the sample sizes of G_v _and G_s _transcripts to be the same as that of the G_a _transcripts. As shown in Figure 3, G_a_-G_0 _and G_v_-G_0 _comparisons have smaller *P*-values (more significant differences) than the G_s_-G_0 _comparisons (*P *< 4.0E-12 by the Kolmogorov-Smirnov Test). Therefore, when the factor of sample size is controlled, the overall result that indels are more depleted downstream of AISs and VuAUGs holds well. Notably, even in the G_s_-G_0 _comparison in the case of pure 5'UTRs (which gives the most conservative estimation), more than 6% of the *P *values are smaller than 0.05 for both human and mouse. This observation suggests that non-3n indels may be subject to weak purifying selection pressure downstream of SuAUGs.

## Discussion

### Possible reasons for non-3n indel depletion in 5'UTRs

We have demonstrated that non-3n indels are subject to purifying selection in mammalian 5'UTRs conditional on the presence of uORFs. We show that both alternative and overlapping uORFs contribute to decreased non-3n indels downstream of their uAUGs, and that strict uORFs have only minor effects in this regard. These results hold well when technical issues in transcript isoform selection, difference in alignment tool, and differences in transcript structures between human and mouse are controlled.

The suppression of non-3n indels downstream of AISs and VuAUGs implies the functional importance of these two uAUG types. Two possible reasons may explain this observation. The first is the functional importance of uORF-associated protein products. Translation of overlapping uORFs always generates radically different peptides from those translated from the main CDS because of the difference in reading frame [27]. Strict uORFs can also produce functional proteins when translated [28,29]. Therefore, additional constraints on non-3n indels unrelated to peptide coding may have separated strict uORFs from the other two types of uORFs. Another explanation is that overlapping uORFs can give rise to in-frame N-truncated peptides, which may have different molecular functions from the original, full-length peptides [17]. Such N-truncated peptides may change in length or simply disappear if non-3n indels occur downstream of VuAUGs. On the other hand, alternative uORFs can lead to the generation of N-extended peptides, which could have different functions from original peptides (e.g. the human regulators of G-protein signalling (RGS2)) [30]. The functional disruption of such N-extended or N-truncated peptides by non-3n indels may be detrimental to the organism in general, and thus could have been removed by natural selection. A recent study provides evidence of the importance of alternative translation start sites by showing that start codons downstream of TISs are evolutionarily conserved [31]. It is suggested that alternative start sites may work as "backup" translational initiation sites and thus may increase the efficiency of translation [31]. The same comment likely also applies to the AISs analyzed in this study.

The second possible explanation for the uAUG-related selection pressure on non-3n indels is the evolutionary conservation of protein abundance. As shown in Figure 1B, non-3n indels may cause interchanges between the three types of uORFs (Figure 1B). In the case of alternative uORFs, downstream non-3n indels can lead to the generation of either strict or overlapping uORFs, causing strong inhibition of protein production of the main CDS in both cases. Such drastic changes in protein abundance are likely deleterious. By contrast, when a non-3n indel occurs downstream of an SuAUG, the affected strict uORF may either become an alternative or overlapping uORF, or remain a strict uORF (but with a different length) (Figure 1B). We suggest that the latter case is more likely, for changing a strict uORF to an alternative or overlapping uORF requires that the reading frame starting from the uAUG remain open until it reaches the TIS. Furthermore, in the case of alternative uORF, the reading frame must be the same as that used by the main CDS. These requirements are difficult to fulfil considering that SuAUGs are relatively distant from the TIS (about 70~75% of the 5'UTR length). As such, non-3n indels that occur downstream of SuAUGs may not lead to changes in uORF type in most cases. Such indels may have no significant effects on changing the protein abundance of the downstream CDS, and thus may have small fitness effects. Lastly, in the case of overlapping uORFs, the occurrence of non-3n indels has a higher possibility of changing them into alternative uORFs than in the case of strict uORFs, because VuAUGs are typically closer to the TIS (about 40% of the 5'UTR length). Overlapping uORFs can result in nearly complete inhibition of protein translation or generation of N-truncated protein [10,18,19]. Furthermore, overlapping uORFs can serve important regulatory roles [10]. Therefore, non-3n indels in overlapping uORFs may be selectively disfavoured. A non-3n indel may also change an overlapping uORF to a strict uORF, or simply change the length of the original uORF (without changing its type). In these cases, non-3n indels may not have significant effects in changing protein abundance, and thus may be tolerated by selection.

One unexpected observation from our results is that four of the six datasets demonstrate lower *P *values in the G_v _transcripts than in the G_a _transcripts (Figure 3), indicating that overlapping uORFs may have contributed stronger constraints on non-3n indels in 5'UTRs than alternative uORFs. That said, the real cause of this G_v_-G_a _difference remains unclear.

Notably, it has been recently reported that 3'UTRs actually have a larger effect on protein abundance than 5'UTRs, which appear to account for ~1% of the variations in protein abundance [32]. How can we observe any selection pressure on non-3n indels in 5'UTRs if these non-coding regions have such a "small" effect on protein abundance? There are three possible explanations. Firstly, as we mentioned above, the non-3n indels in 5'UTRs may affect both the abundance and the peptide sequence of the affected gene. The "qualitative" change may also be subject to selection pressure. Secondly, even though on average 5'UTRs account for only a small proportion of the variations in protein abundance genome-widely, in individual genes the variations can be very large, as was demonstrated by a recent study [16]. The cases where 5'TURs have very small effects on protein abundance may actually add to the noise in our analysis. However, we have found clear signals of selection pressure on non-3n indels despite these potential noises, which in fact reflects the strength of the "real" signals. Finally, the uORFs *per se *may be biologically functional, in terms of either their peptide products or their regulatory roles. The disruption of functional uORFs is thus likely subject to selective constraint.

### Limitations of the study

The current analysis contains a few limitations. Firstly, determining which transcript of a gene to analyze is controversial. This study used three different criteria for transcript selection. In the case of the transcripts with the longest 5'UTRs, one uORF may partly overlap with CDS. In this case, the ISI value may be smaller than expected because of the strong constraint on reading frame preservation in coding sequences. In addition, the classification of the uORFs (alternative, strict, or overlapping) in these 5'UTRs is sometimes ambiguous (see Additional file 3 for an example). Analyzing the transcripts with pure 5'UTRs avoids this problem. However, this practice will lead to significantly decreased numbers of uORFs and severely reduced sample sizes, which in turn may result in decreased statistic power and potential sampling biases. These two criteria for transcript selection represent two extremes. The results derived using randomly selected transcripts fall in-between. Nevertheless, this study obtained consistent results across all datasets, indicating that depletion of non-3n indels is unlikely to result solely from constraints in the main coding sequences.

Secondly, since our study is based on pairwise sequence alignments, we cannot distinguish between insertions and deletions, nor can we infer the lineage specificity of the identified indels. We do not know exactly in which lineage the indels have affected the structures of the 5'UTRs (i.e. the types of uORFs). This is important because the 5'UTRs of human and mouse transcripts may have different lengths and uORFs. When a non-3n indel occurs to a lineage whose transcript does not contain any uORFs or 5'UTR exons, this indel adds to the noise in our analysis. We cannot eliminate such noises without using multiple-species sequence alignments (which, however, will dramatically decrease the sample size and render the analysis infeasible). To overcome this problem, this study performed analyses using the transcript structures of human and mouse separately. The results from both species turn out to be consistent with each other. Therefore, in spite of the above limitation, our results seem to have revealed a biological fact.

## Conclusions

To the best of our knowledge, this is the first study to demonstrate the selective constraint on non-3n indels in 5'UTRs. This constraint may result from the requirement to preserve either the translational regulatory elements (uORFs) in 5'UTRs or the functions of the peptides whose translation is associated with uORFs. Our results thus demonstrate the impacts of indels in the evolution of 5'UTRs in mammalian genomes and re-assure the functional importance of uORFs from a different angle.

## Methods

### Data collection

In this study, we analyze the human and mouse transcripts because their genomes have been fully sequenced and extensively curated [33,34]. In addition, the transcriptomes of these two species have been well characterized. The annotations for 5'UTRs are thus fairly accurate for the two species. The sequences of experimentally verified transcripts with known protein products of one-to-one human-mouse orthologous genes, based on the Ensembl release 54 http://www.ensembl.org, were retrieved through BioMart [35]. Non-protein-coding genes and protein-coding genes whose transcripts did not contain 5'UTRs were excluded. Potentially misannotated transcripts (whose locations of TISs were inconsistent with that observed in the DNA sequences) were also excluded. To avoid repetitive counts of the same indels, only one transcript was selected for each gene by three different criteria: (1) a randomly selected transcript, (2) the transcript with the longest 5'UTR, and (3) the transcript with a "pure" 5'UTR. A pure 5'UTR is one that does not overlap with any coding sequences in other splicing isoforms (See Additional file 4 for an example).

### Sequence alignments and identification of indels and uORFs

Indels were identified based on the human-mouse pairwise genomic sequence alignments downloaded from the University of California, Santa Cruz (UCSC) Genome Browser http://genome.ucsc.edu/ [36]. The versions of the human and mouse genomes (hg18 and mm9, respectively) correspond to Ensembl release 54.

To ensure that human-mouse orthologous sequences were compared in our study, this work only retained the alignments that include one-to-one human-mouse orthologous genes annotated by Ensembl. In addition, to avoid mis-assignment of gaps (indels), the alignable exonic sequences in one species are required to overlap with > 80% of the annotated exonic sequences of the other species. The alignments must cover the entirety of the annotated 5'UTRs. Consequently, this study obtained ~6,000 human and mouse genes for subsequent analyses (Table 1). To examine whether different alignment tools affect the overall results, the Pecan alignment program [26] was used with default parameters to re-align the retrieved human-mouse orthologous sequences.

The 5'UTRs of the retrieved transcripts were then scanned for the existence of uAUGs. Around half of the analyzed human and mouse transcripts were found to have at least one uAUG (Additional file 5). These proportions are similar to those observed in previous studies [16,24]. Here, a uORF is defined as a putative open reading frame in 5'UTR starting with a uAUG and composed of at least nine nucleotides, including the stop codon. Human and mouse orthologous genes may have different 5'UTR structures and different numbers and types of uORFs. Therefore, we performed our analyses according to the human and mouse gene annotations separately.

### Measurement of selection pressure on indel lengths -- the indel selection index

To evaluate the evolutionary constraints on indel lengths in different parts of a 5'UTR, we defined(1)

where *N *represents the number of indels, the subscripts "*n3n*" and "*3n*" represent non-3n and 3n indels. The subscripts "*d*" and "*u*" indicate the 5'UTR regions downstream and upstream of the uAUGs (or reference points) of interest. Adding the pseudocount 0.5 ensures that the denominator is not zero. Using different pseudocounts changes the magnitude, but not the sign (positive or negative) of the *R *value (Additional file 6). The ratio of non-3n to 3n indels upstream of uAUGs serves as the "background" to measure the depletion (or enrichment) of non-3n indels downstream of uAUGs.

Theoretically, if selection has no preference on indel sizes across 5'UTRs, *R *should be equal to zero. However, when non-3n indels downstream of uAUGs are suppressed by purifying selection, *R *should be smaller than 0. The statistical power of *R *is expected to increase with the number of indels. Yet in our dataset, the lengths of more than 90% of the 5'UTRs are shorter than 1 kb (and thus the numbers of indels are small). The relatively small sample sizes may lead to unexpected biases. To address this issue, we developed the "Indel Selection Index" (ISI) to measure the probability that an *R *value is smaller than the random expectation. The ISI is defined as(2)

which represents the proportion of the randomized *R*-values (*R_shuffled_*) that is smaller than the observed *R*-value. The distribution of *R_shuffled _*was generated by randomly shuffling the locations of the indels 1,000 times for each transcript, while retaining the lengths and numbers of indels of the 5'UTR. An ISI value smaller than the neutral expectation indicates depletion of non-3n indels downstream of a uAUG. The neutral distribution of ISIs were derived from the transcripts without uAUGs (see Additional file 1), with the upstream/downstream differentiation point moving from the cap to TIS by an increment of 1% of 5'UTR length. Note that we use ISI rather than comparing the non-3n to 3n indel ratios between 5'UTR and intergenic/intronic regions to control for the potential biological differences between 5'UTR and the other noncoding regions.

## Authors' contributions

CHC carried out all of the analyses. BYL and FCC conceived the study. CHC, BYL and FCC designed the study and drafted the manuscript. All authors have read and approved the final manuscript.

## Supplementary Material

Additional file 1 **The indel selection index in 5'UTRs without uAUGs**. The X-axis indicates the relative distance from the 5'-cap to the reference point in terms of percentage of the 5'UTR length. The results were derived from the genes with randomly selected transcripts. Click here for file

Additional file 2 **Distributions of ISI values according to Pecan-aligned sequences, with reference to the gene annotations of (A) human; and (B) mouse**. The numbers in the parentheses following G_0 _indicate the median distances of the uAUGs from 5' cap in terms of percentage of 5'UTR length in the non-G_0 _transcripts. These proportions of length are referenced to determine which G_0 _distributions to use in the comparisons. The *P *values of pair-wise differences (calculated by using the Mann-Whitney *U *test) are shown at the top. The symbols "*", "**", and "***" represent 0.01 ≦ *P *< 0.05, 0.001 ≦ *P *< 0.01, and *P *< 0.001, respectively. Click here for file

Additional file 3 **The ISI distributions of G_v _transcripts with different uAUG locations**. Each **G**_**V **_dataset is divided into three equal-sized subgroups according to the relative locations of uAUGs. The numbers in the parentheses following G_0 _indicate the median distances of the uAUGs from 5' cap in terms of percentage of 5'UTR length in the **G**_**V **_transcripts. The *P *values (by the Mann-Whitney U test) for the ISI differences between **G**_**V **_and the corresponding G_0 _transcripts are shown at the top. Click here for file

Additional file 4 **The five alternatively spliced transcript isoforms of human gene ENSG00000119125**. Only the 5' proximal regions of the transcripts are shown here. Among the isoforms, ENST00000358399 is the only one that contains a pure 5'UTR, which includes no uAUGs at all. Note that this pure 5'UTR is also the shortest one. In comparison, ENST00000238018 contains the longest 5'UTR, which encompasses as many as 15 uAUGs. The exons are represented as blue bars (not to the scale). The black, red, green, and blue inverted triangles represent translation initiation sites, AISs ("A"), VuAUGs ("V"), and SuAUGs ("S"), respectively. Click here for file

Additional file 5 **The numbers and percentages of transcripts analyzed in this study**. "G_am_", "G_sm_", and "G_vm_" indicate transcripts with multiple AISs, SuAUGs, and VuAUGs, respectively. In the G_m _group, more than one type of uORF is found in the 5'UTRs. The subscripts "a", "s", and "v", indicate the presence of AIS, SuAUG, and VuAUG, respectively. Note that only G_a_, G_s_, and G_v _are analyzed in this study. Click here for file

Additional file 6 **The distributions of R values using different pseudocounts**. "c" stands for the pseudocount. The black bar represents a 5'UTR, where solid and open circles indicate the locations of non-3n and 3n indels, respectively. The reference point is used to differentiate the upstream and downstream region of a 5'UTR. Click here for file
