# Supplementary material for: Exploring the selective constraint on the sizes of insertions and deletions in 5' untranslated regions in mammals
Source: BMC Evol Biol. 2011 Jul 5;11:192. doi: 10.1186/1471-2148-11-192 (PMC3146882; doi:10.1186/1471-2148-11-192)
Supplement: Additional file 2 — Distributions of ISI values according to Pecan-aligned sequences, with reference to the gene annotations of (A) human; and (B) mouse. The numbers in the parentheses following G0 indicate the median distances of the uAUGs from 5' cap in terms of percentage of 5'UTR length in the non-G0 transcripts. These proportions of length are referenced to determine which G0 distributions to use in the comparisons. The P values of pair-wise differences (calculated by using the Mann-Whitney U test) are shown at the top. The symbols "*", "**", and "***" represent 0.01 ≦ P < 0.05, 0.001 ≦ P < 0.01, and P < 0.001, respectively. [file 1471-2148-11-192-S2.DOC]

| Randomly selected 5’UTR | Longest 5’UTR | Pure 5’UTR |
| --- | --- | --- |
|  |  |  |

(A)

| Randomly selected 5’UTR | Longest 5’UTR | Pure 5’UTR |
| --- | --- | --- |
|  |  |  |

(B)

Additional file 2 - Distributions of ISI values according to Pecan-aligned sequences, with reference to the gene annotations of (A) human; and (B) mouse.
