# Supplementary material for: Exploring the selective constraint on the sizes of insertions and deletions in 5' untranslated regions in mammals
Source: BMC Evol Biol. 2011 Jul 5;11:192. doi: 10.1186/1471-2148-11-192 (PMC3146882; doi:10.1186/1471-2148-11-192)
Supplement: Additional file 3 — The ISI distributions of Gv transcripts with different uAUG locations. Each GV dataset is divided into three equal-sized subgroups according to the relative locations of uAUGs. The numbers in the parentheses following G0 indicate the median distances of the uAUGs from 5' cap in terms of percentage of 5'UTR length in the GV transcripts. The P values (by the Mann-Whitney U test) for the ISI differences between GV and the corresponding G0 transcripts are shown at the top. [file 1471-2148-11-192-S3.PDF]

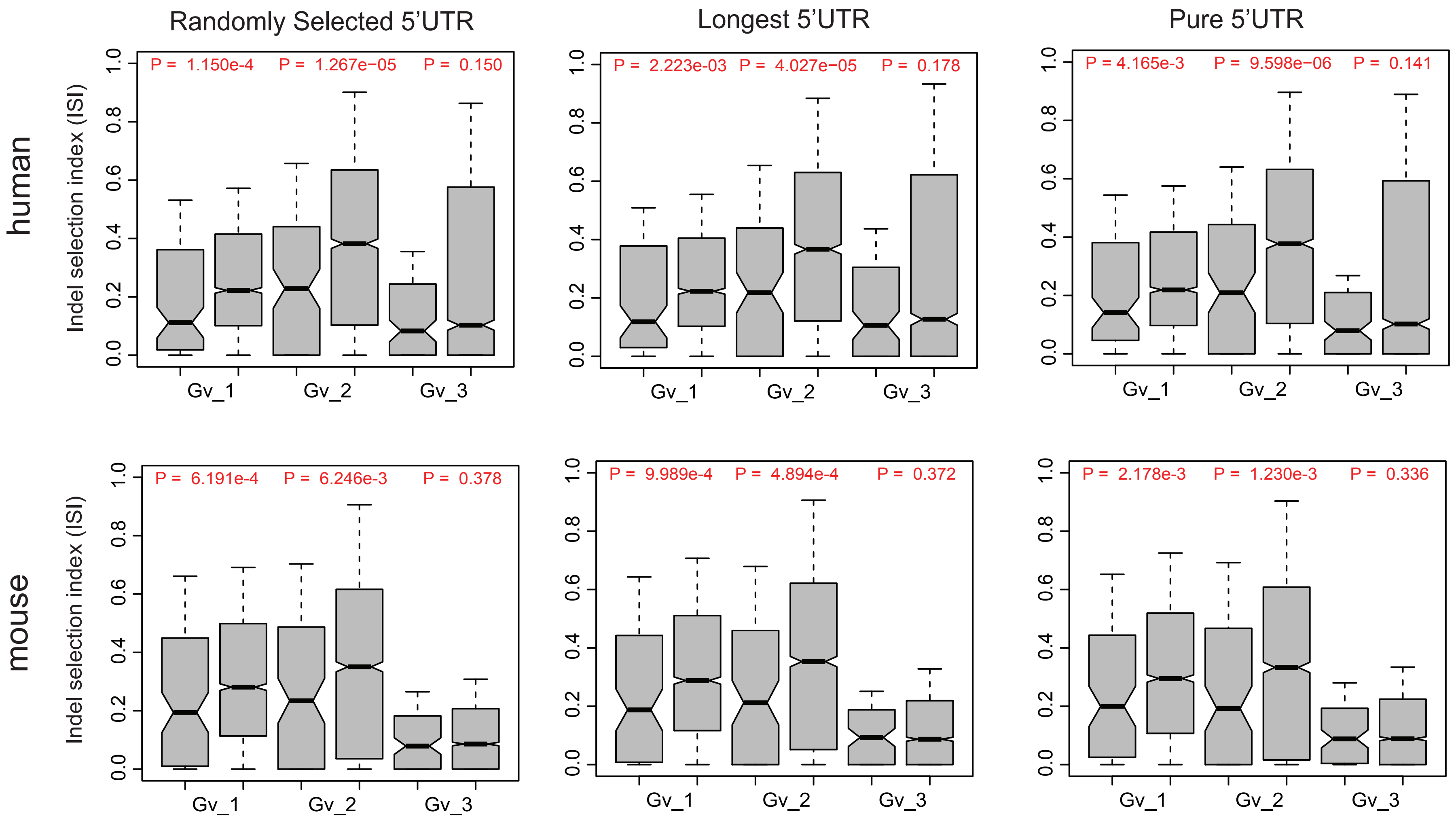


Additional file 3 – The ISI distributions of Gv transcripts with different uAUG locations.
Each GV dataset is divided into three equal-sized subgroups according to the relative locations of uAUGs. The *P* values for the ISI differences between GV and the corresponding G0 transcripts are shown at the top.
