# Supplementary material for: Exploring the selective constraint on the sizes of insertions and deletions in 5' untranslated regions in mammals
Source: BMC Evol Biol. 2011 Jul 5;11:192. doi: 10.1186/1471-2148-11-192 (PMC3146882; doi:10.1186/1471-2148-11-192)
Supplement: Additional file 4 — The five alternatively spliced transcript isoforms of human gene ENSG00000119125. Only the 5' proximal regions of the transcripts are shown here. Among the isoforms, ENST00000358399 is the only one that contains a pure 5'UTR, which includes no uAUGs at all. Note that this pure 5'UTR is also the shortest one. In comparison, ENST00000238018 contains the longest 5'UTR, which encompasses as many as 15 uAUGs. The exons are represented as blue bars (not to the scale). The black, red, green, and blue inverted triangles represent translation initiation sites, AISs ("A"), VuAUGs ("V"), and SuAUGs ("S"), respectively. [file 1471-2148-11-192-S4.DOC]

Additional file 4 - The five alternatively spliced transcript isoforms of human gene ENSG00000119125. Only the 5’ proximal regions of the transcripts are shown here. Among the isoforms, ENST00000358399 is the only one that contains a pure 5’UTR, which includes no uAUGs at all. Note that this pure 5’UTR is also the shortest one. In comparison, ENST00000238018 contains the longest 5’UTR, which encompasses as many as 15 uAUGs. The exons are represented as blue bars (not to the scale). The black, red, green, and blue inverted triangles represent translation initiation sites, AISs (“A”), VuAUGs (“V”), and SuAUGs (“S”), respectively.
