# Supplementary material for: Exploring the selective constraint on the sizes of insertions and deletions in 5' untranslated regions in mammals
Source: BMC Evol Biol. 2011 Jul 5;11:192. doi: 10.1186/1471-2148-11-192 (PMC3146882; doi:10.1186/1471-2148-11-192)
Supplement: Additional file 5 — The numbers and percentages of transcripts analyzed in this study. "Gam", "Gsm", and "Gvm" indicate transcripts with multiple AISs, SuAUGs, and VuAUGs, respectively. In the Gm group, more than one type of uORF is found in the 5'UTRs. The subscripts "a", "s", and "v", indicate the presence of AIS, SuAUG, and VuAUG, respectively. Note that only Ga, Gs, and Gv are analyzed in this study. [file 1471-2148-11-192-S5.DOC]

Additional file 5 - The numbers and percentages of transcripts analyzed in this study.

| No. uAUGs | Criteria | | Randomly-selected 5’UTR | | | | Longest 5’UTR | | | | Pure 5’UTR | | | |
| --- | --- | --- | --- | --- | --- | --- | --- | --- | --- | --- | --- | --- | --- | --- |
|  | Species | | Human | | Mouse | | Human | | Mouse | | Human | | Mouse | |
| 0 | G_0_ | | 3,265 | (54.0%) | 3,560 | (58.9%) | 2,701 | (46.6%) | 3,153 | (54.4%) | 3,144 | (55.2%) | 3,368 | (59.1%) |
| 1 | G_a_ | | 59 | (1.0%) | 55 | (0.9%) | 80 | (1.4%) | 70 | (1.2%) | 35 | (0.6%) | 37 | (0.6%) |
|  | G_s_ | | 759 | (12.6%) | 709 | (11.7%) | 709 | (12.2%) | 682 | (11.8%) | 780 | (13.7%) | 733 | (12.9%) |
|  | G_v_ | | 348 | (5.8%) | 341 | (5.6%) | 307 | (5.3%) | 295 | (5.1%) | 334 | (5.9%) | 310 | (5.4%) |
| ≧2 | G_am_ | | 14 | (0.2%) | 6 | (0.1%) | 19 | (0.3%) | 6 | (0.1%) | 3 | (0.1%) | 3 | (0.1%) |
|  | G_sm_ | | 799 | (13.2%) | 747 | (12.4%) | 855 | (14.7%) | 805 | (13.9%) | 858 | (15.1%) | 790 | (13.9%) |
|  | G_vm_ | | 53 | (0.9%) | 44 | (0.7%) | 49 | (0.8%) | 38 | (0.7%) | 46 | (0.8%) | 35 | (0.6%) |
|  | G_m_ | G_as_ | 148 | (2.4%) | 103 | (1.7%) | 294 | (5.1%) | 190 | (3.3%) | 45 | (0.8%) | 37 | (0.6%) |
|  |  | G_av_ | 32 | (0.5%) | 27 | (0.4%) | 34 | (0.6%) | 35 | (0.6%) | 7 | (0.1%) | 13 | (0.2%) |
|  |  | G_sv_ | 469 | (7.8%) | 387 | (6.4%) | 539 | (9.3%) | 413 | (7.1%) | 425 | (7.5%) | 357 | (6.3%) |
|  |  | G_asv_ | 100 | (1.7%) | 67 | (1.1%) | 213 | (3.7%) | 113 | (1.9%) | 23 | (0.4%) | 17 | (0.3%) |
|  |  | Σ | 6,046 |  | 6,046 |  | 5,800 |  | 5,800 |  | 5,700 |  | 5,700 |  |

“G_am_”, “G_sm_”, and “G_vm_” indicate transcripts with multiple AISs, SuAUGs, and VuAUGs, respectively. In the G_m_ group, more than one type of uORF is found in the 5’UTRs. The subscripts “a”, “s”, and “v”, indicate the presence of AIS, SuAUG, and VuAUG, respectively. Note that only G_a_, G_s_, and G_v_ are analyzed in this study.
