# Supplementary material for: Exploring the selective constraint on the sizes of insertions and deletions in 5' untranslated regions in mammals
Source: BMC Evol Biol. 2011 Jul 5;11:192. doi: 10.1186/1471-2148-11-192 (PMC3146882; doi:10.1186/1471-2148-11-192)
Supplement: Additional file 6 — The distributions of R values using different pseudocounts. "c" stands for the pseudocount. The black bar represents a 5'UTR, where solid and open circles indicate the locations of non-3n and 3n indels, respectively. The reference point is used to differentiate the upstream and downstream region of a 5'UTR. [file 1471-2148-11-192-S6.DOC]

**Distance from the 5’ cap to the reference point**

**(% of the 5’UTR length)**

Additional file 6 - The distributions of *R* values using different pseudocounts (c). The black bar represents a 5’UTR, where solid and open circles indicate the locations of non-3n and 3n indels, respectively. The reference point is used to differentiate the upstream and downstream region of a 5’UTR.
